# Supplementary figures and images for: Ancient segmentally duplicated LCORL retrocopies in equids
Source: PLoS One. 2023 Jun 8;18(6):e0286861. doi: 10.1371/journal.pone.0286861 (PMC10249811; doi:10.1371/journal.pone.0286861)

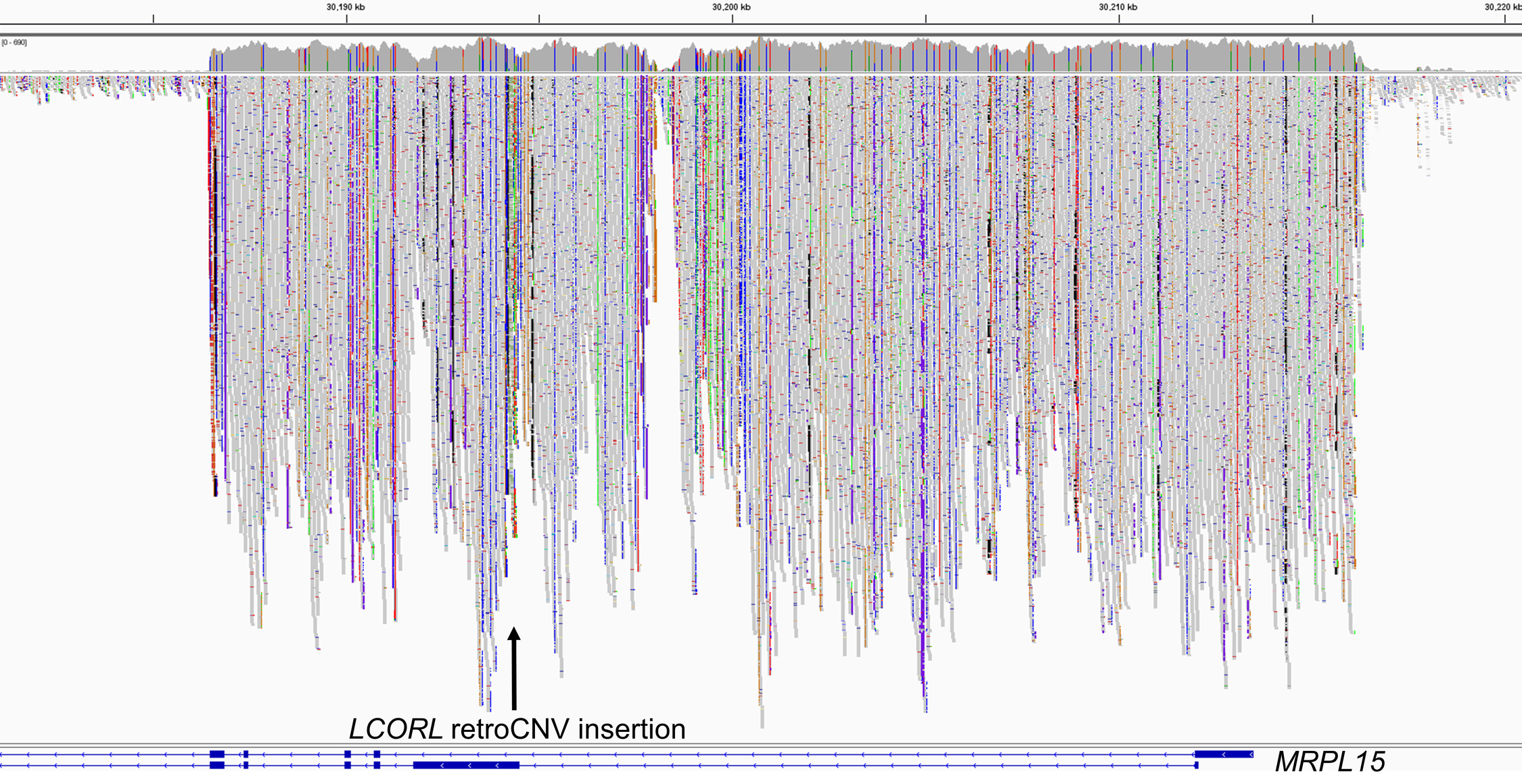

Supplement: S1 Fig — A segmental duplication encompassing the MRPL15 gene on chromosome 9 contains the LCORL retrocopy insertion at chr9:30194359–30194380. Colors highlight the presence of SNV and indels within the duplication. (TIF) [file pone.0286861.s001.tif]

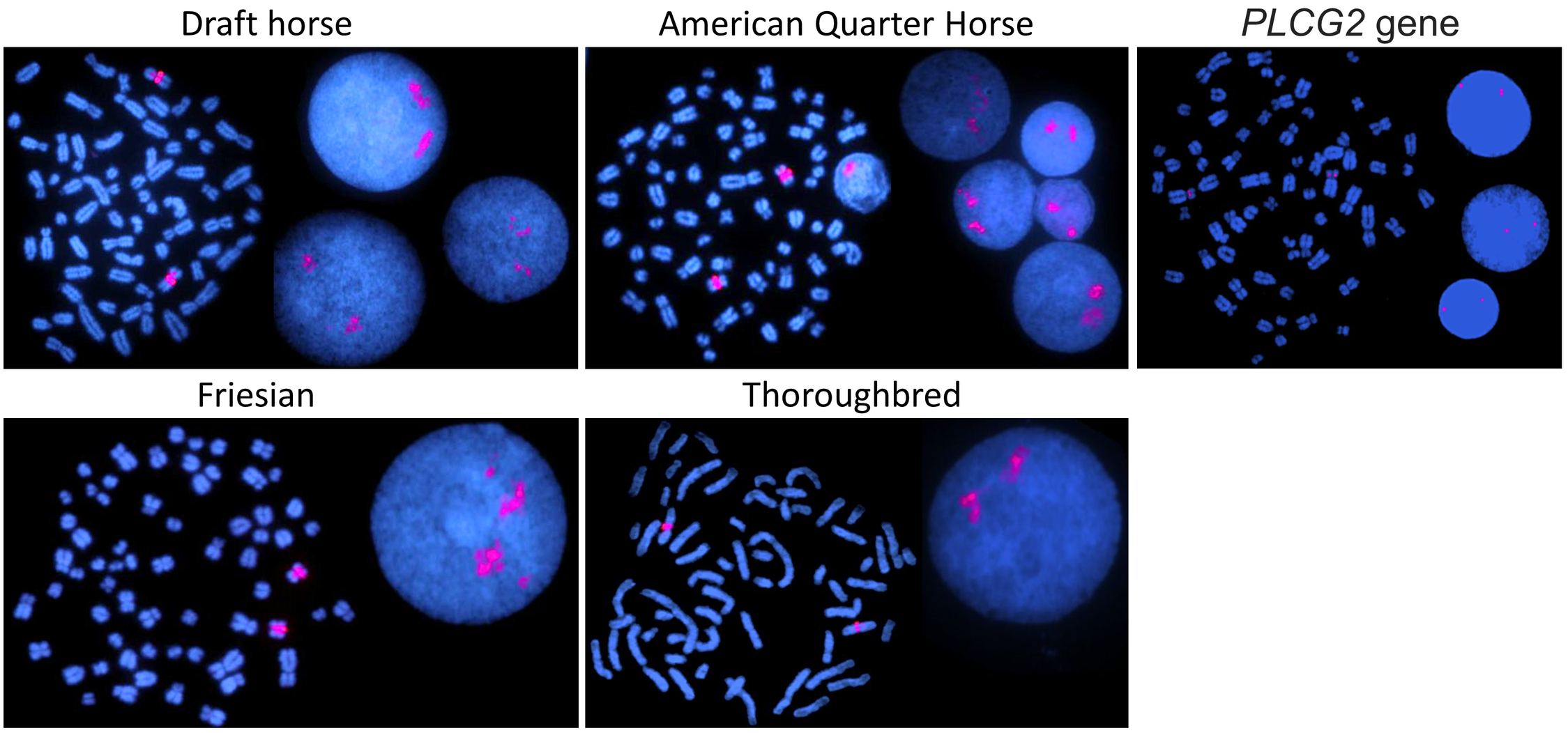

Supplement: S2 Fig — FISH with LCORL retrocopies containing horse BAC clone 164B3 on metaphase and interphase chromosomes of four different horse breeds shows LCORL copy number variation between individuals. FISH using the horse BAC clone 79K4 containing the single copy gene PLCG2 are shown for contrast. (TIF) [file pone.0286861.s002.tif]

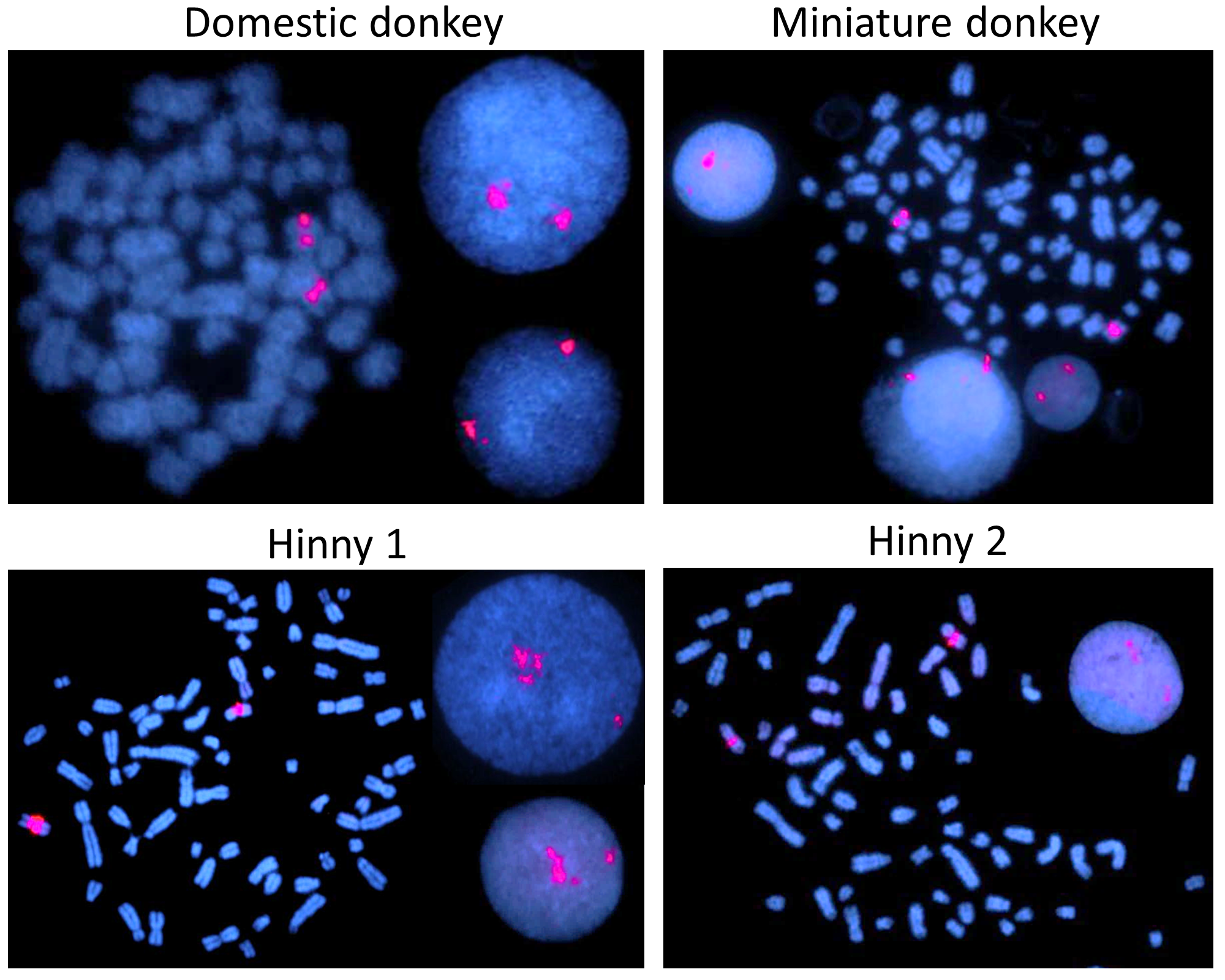

Supplement: S3 Fig — FISH with LCORL retrocopies containing horse BAC clone 164B3 on metaphase and interphase chromosomes of two donkeys and two hinnies showing LCORL copy number variation between individual donkeys and the donkey and the horse. Note that in the hinnies, the chromosomes with less copies is the donkey chr12 and the chromosome with more copies is the horse chr9. (TIF) [file pone.0286861.s003.tif]

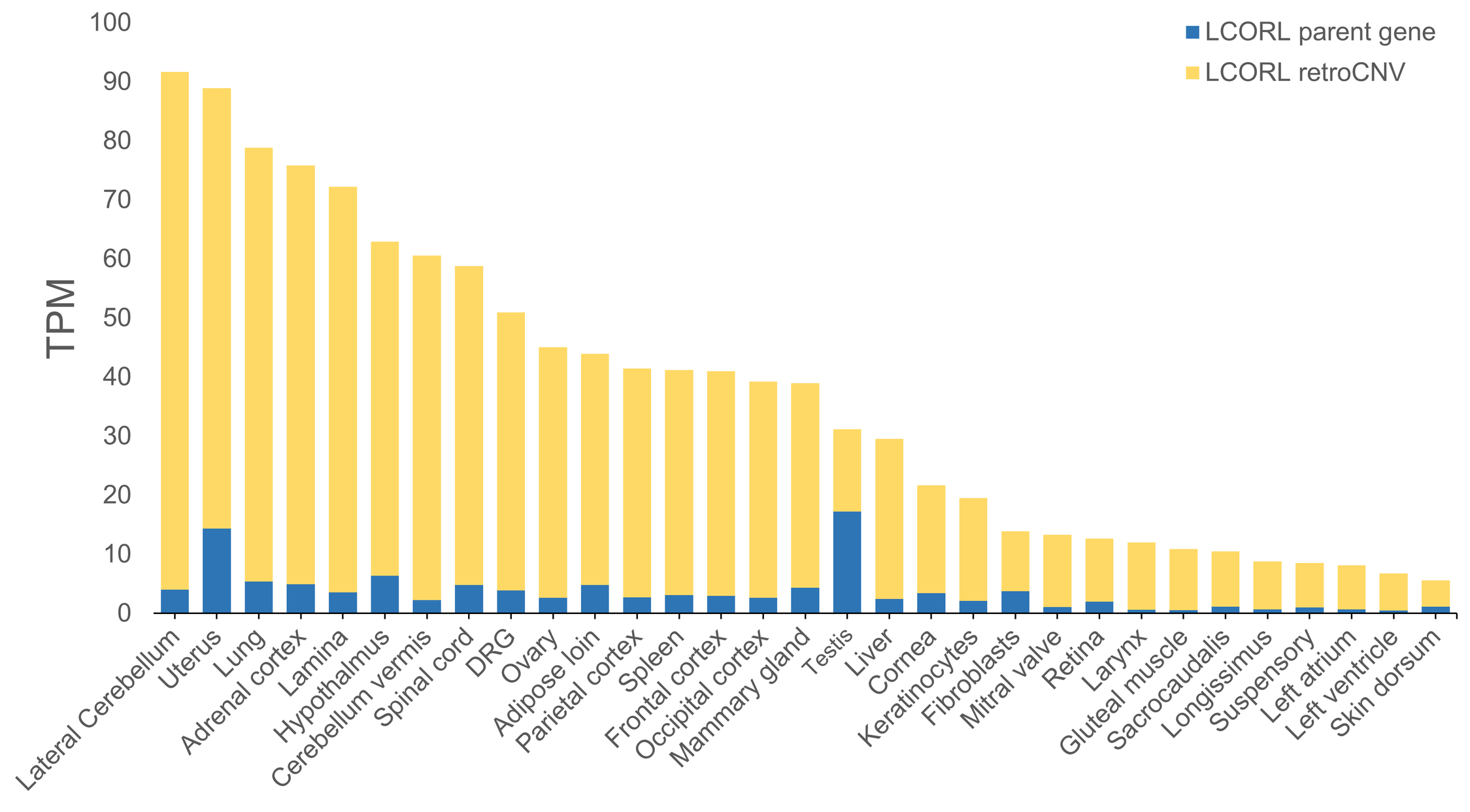

Supplement: S4 Fig — Percentages of total LCORL transcripts derived from the retroCNV is shown for each tissue type. The LCORL retrocopy comprised the majority of overall LCORL transcripts in all tissue types except testis. (TIF) [file pone.0286861.s004.tif]

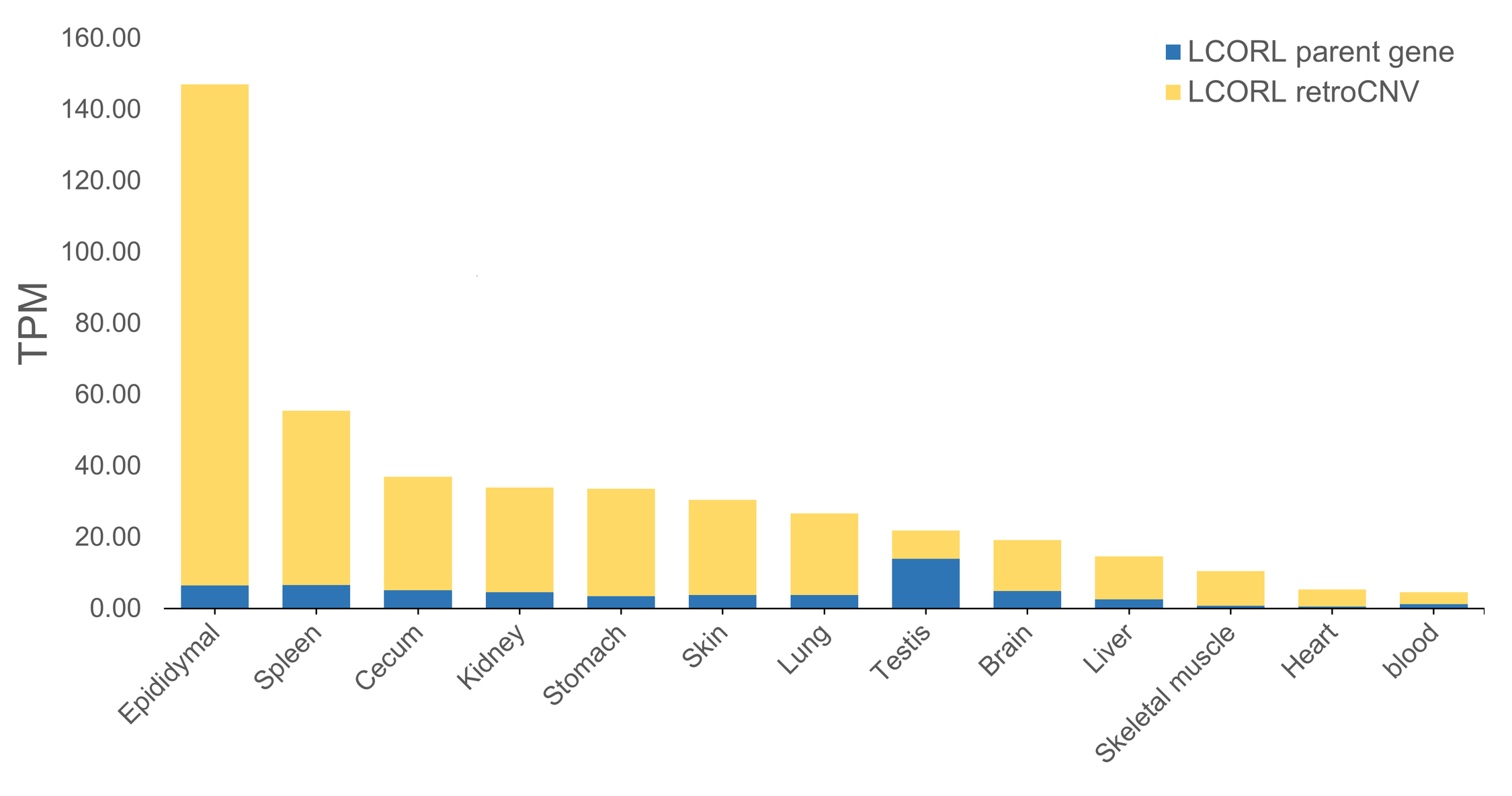

Supplement: S5 Fig — The LCORL retrocopy comprised the majority of overall LCORL transcripts in all tissue types except testis. (TIF) [file pone.0286861.s005.tif]

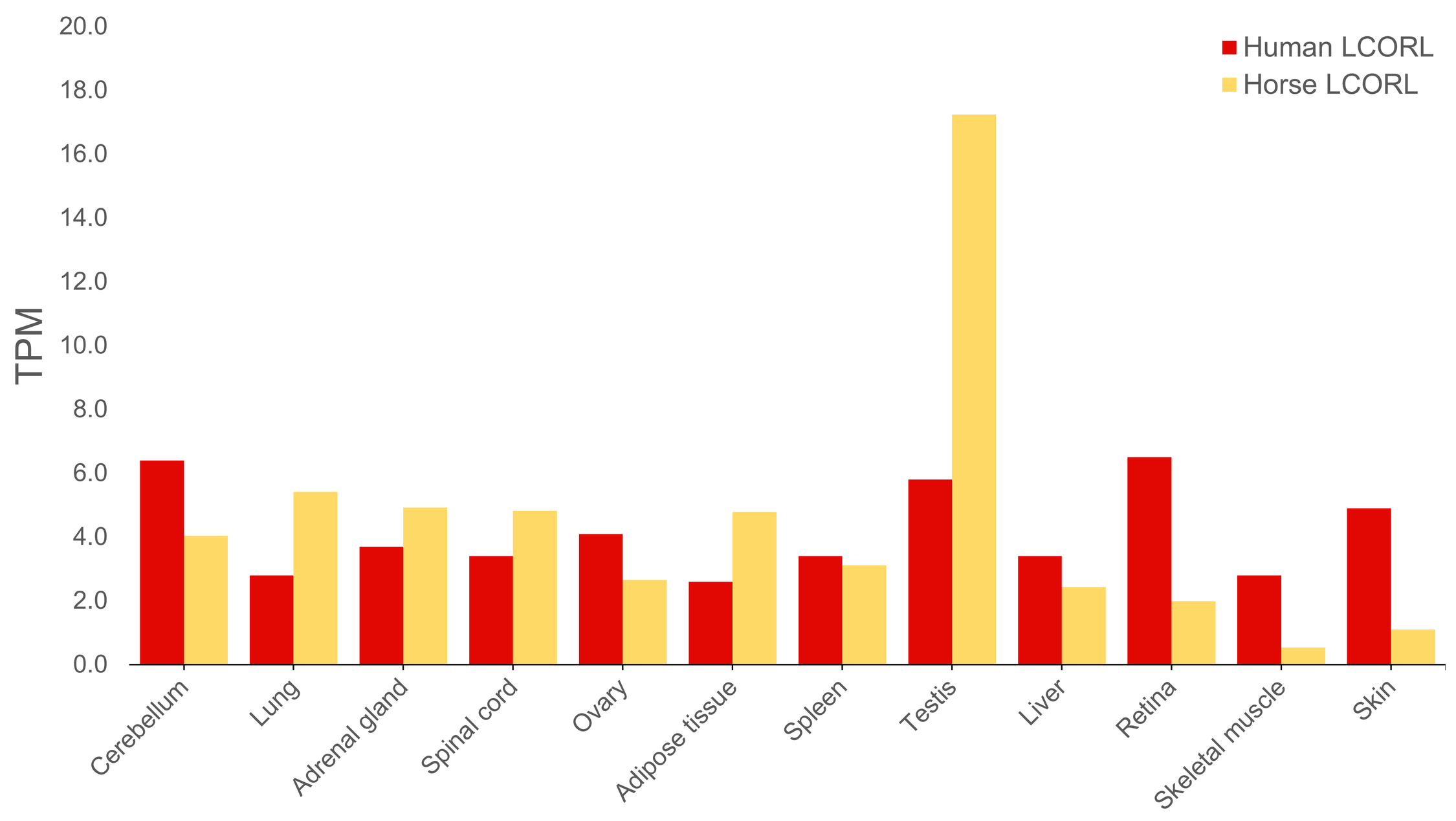

Supplement: S6 Fig — Expression of the parent gene in horses is comparable to human LCORL across tissue types. (TIF) [file pone.0286861.s006.tif]

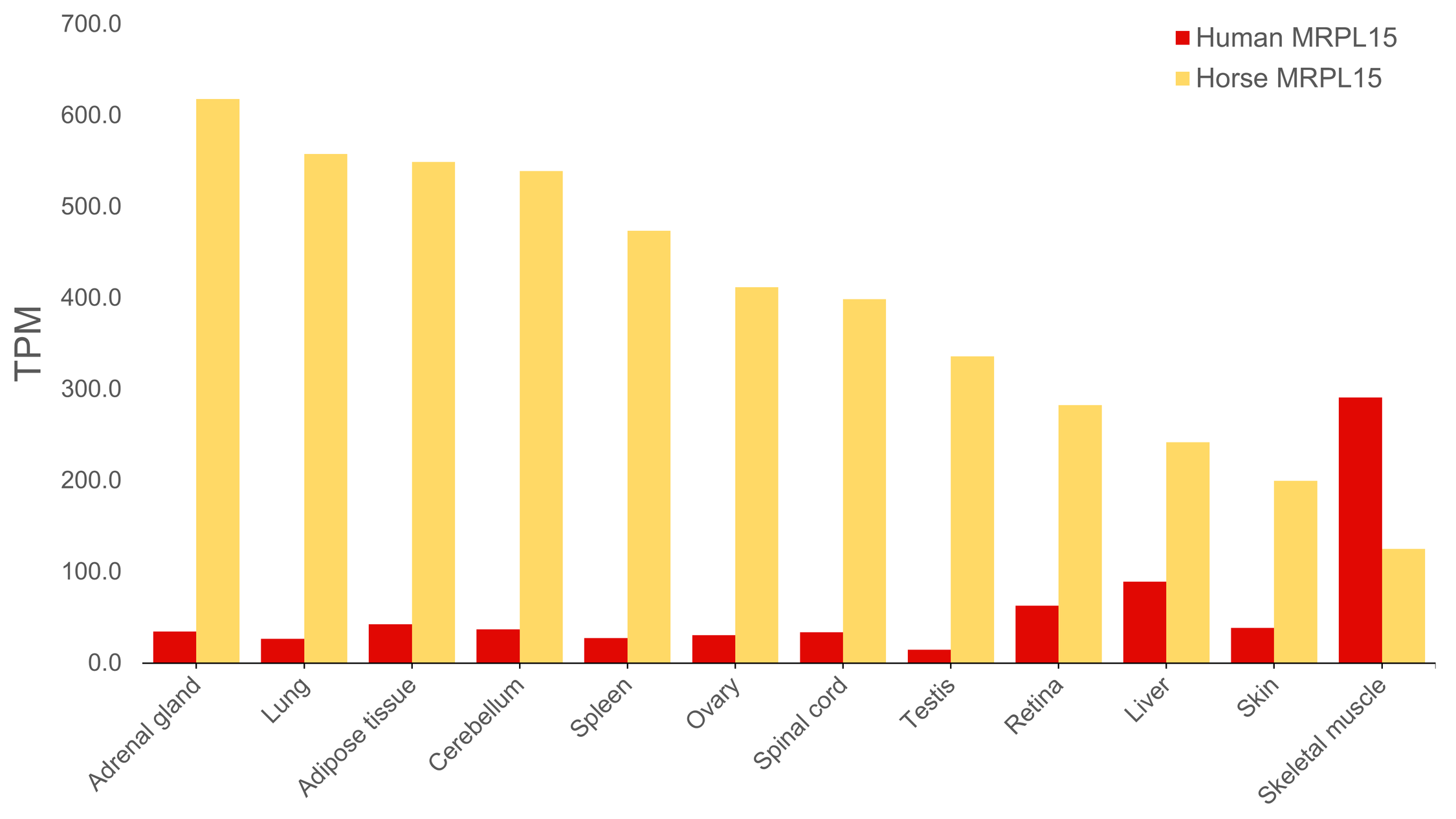

Supplement: S7 Fig — Expression of the segmentally duplicated MRPL15 gene is increased in horses relative to humans. (TIF) [file pone.0286861.s007.tif]

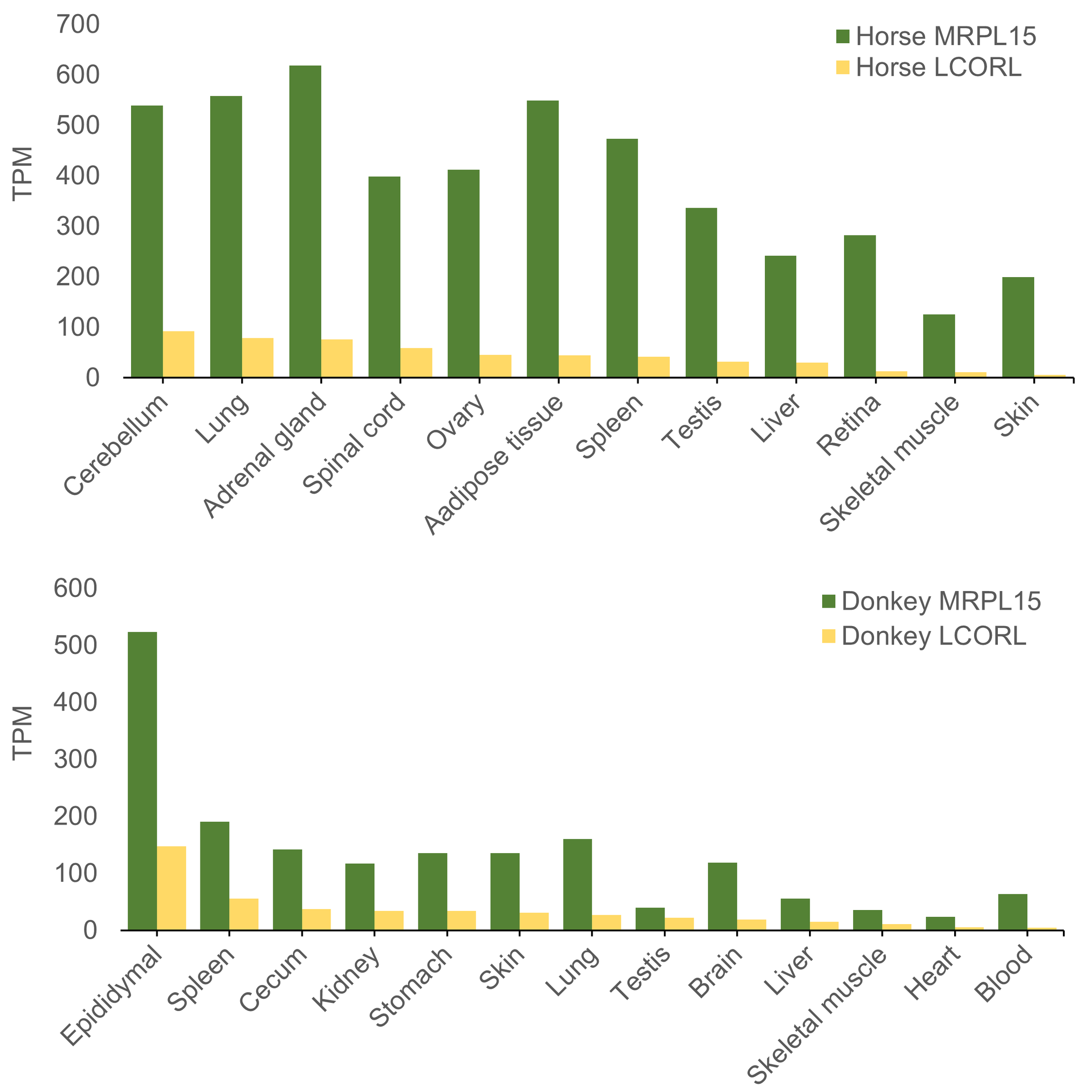

Supplement: S8 Fig — (TIF) [file pone.0286861.s008.tif]

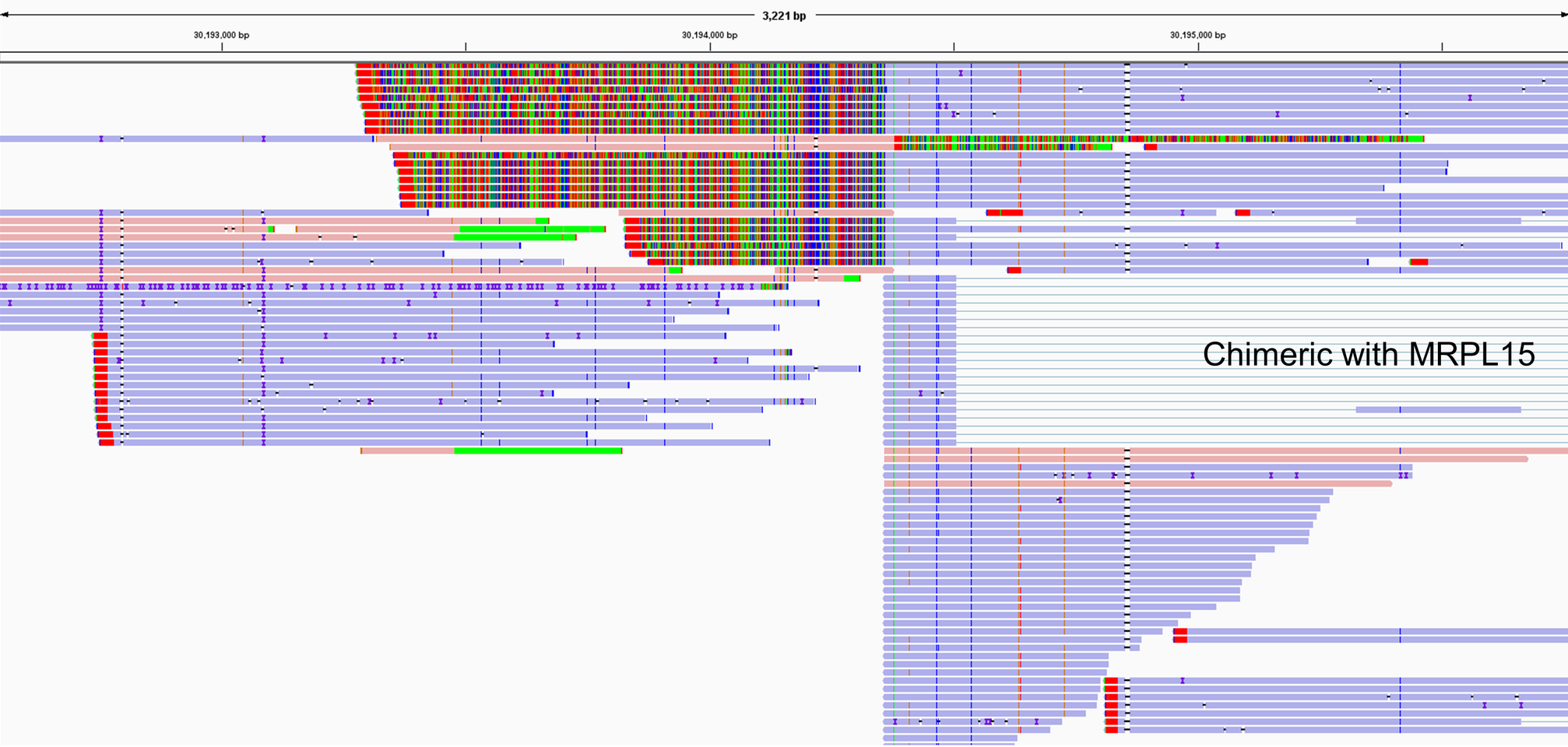

Supplement: S9 Fig — The soft-clipped bases at the insertion site indicate sequences which match the LCORL parent gene on chr3. Chimeric reads were observed between the LCORL retrocopy and the nearby MRPL15 gene. Antisense reads highlighted in red were also observed at the insertion site. (TIF) [file pone.0286861.s009.tif]

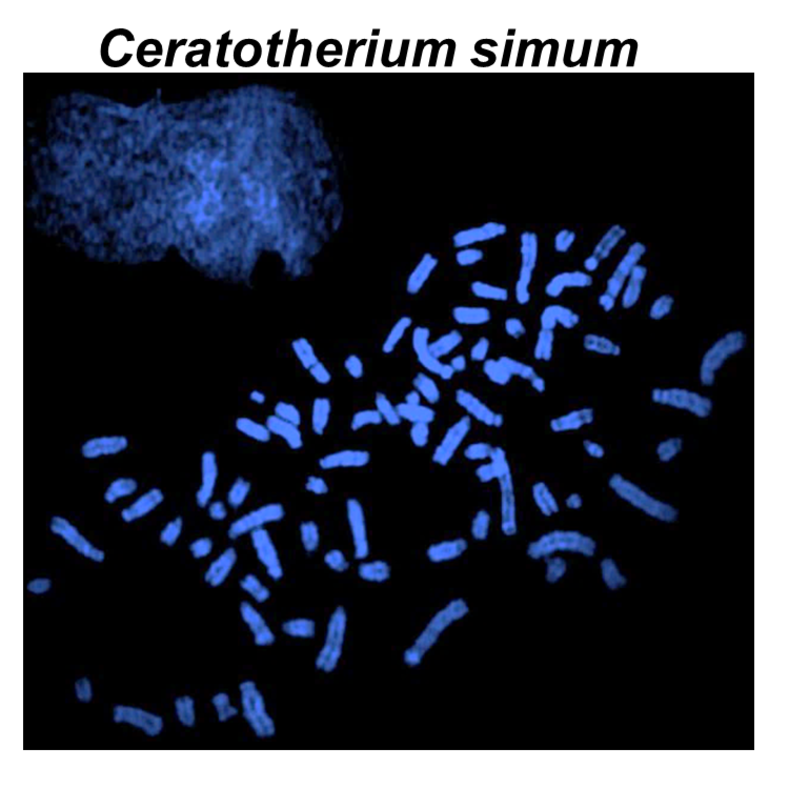

Supplement: S10 Fig — Hybridization signals were absent in the rhino, indicating the absence of LCORL retrocopies. (TIF) [file pone.0286861.s010.tif]
